# Crossroads in Liver Transplantation: Is Artificial Intelligence the Key to Donor–Recipient Matching?

**DOI:** 10.3390/medicina58121743

**Published:** 2022-11-28

**Authors:** Rafael Calleja Lozano, César Hervás Martínez, Francisco Javier Briceño Delgado

**Affiliations:** 1 Liver Transplantation Unit, General and Digestive Surgery Department, Reina Sofía University Hospital, 14004 Cordoba, Spain; 2 GC18 Translational Research in Solid Organ Transplantation Surgery—Maimonides Biomedical Research Institute (IMIBIC), 14004 Cordoba, Spain; 3 Department of Computer Sciences and Numerical Analysis, Universidad de Córdoba, 14014 Cordoba, Spain

**Keywords:** donor–recipient matching, artificial intelligence, deep learning, artificial neural networks, random forest, liver transplantation outcomes

## Abstract

Liver transplantation outcomes have improved in recent years. However, with the emergence of expanded donor criteria, tools to better assist donor–recipient matching have become necessary. Most of the currently proposed scores based on conventional biostatistics are not good classifiers of a problem that is considered “unbalanced.” In recent years, the implementation of artificial intelligence in medicine has experienced exponential growth. Deep learning, a branch of artificial intelligence, may be the answer to this classification problem. The ability to handle a large number of variables with speed, objectivity, and multi-objective analysis is one of its advantages. Artificial neural networks and random forests have been the most widely used deep classifiers in this field. This review aims to give a brief overview of D–R matching and its evolution in recent years and how artificial intelligence may be able to provide a solution.

## 1. Introduction

The problem of liver donor–recipient (D–R) matching is not new and is inherent to organ transplantation. The improvement in surgical techniques, postoperative management, diagnosis, and treatment of post-transplant complications or the development of liver preservation techniques are some of the barriers that liver transplantation has overcome in recent years. As a result, the recipient- and graft-survival rates at one year are above 95%, and long-term survival has become the norm. However, the Achilles’ heel of liver transplantation continues to be the disproportionate numbers between the number of donors and the number of waitlisted patients. This leads to long waiting times and mortality among patients waiting for a graft that can reach as high as 20% in certain patient groups [1,2]. Far from improving this situation, the current expansion of inclusion criteria, such as elderly recipients or indications for malignant tumors (transplant oncology), may further aggravate this problem. This shortage might lead us to think that the use of grafts would be higher. Paradoxically, however, in countries where deceased donor rates are low, the utilization rate is high and inverse [2]. Some of the proposed solutions have been to include expanded criteria donors (ECDs) or improve graft utilization using preservation machines, one of the most promising developing fields [3].

The imbalance between candidates and grafts is further complicated by organ allocation policies since there exist as many policies as decisions about what to prioritize. On the one hand, policies based on the principle of urgency (the “sickest-first” principle) benefit high-risk candidates. On the other hand, policies based on the principles of “individual transplant benefit” and “population-based transplant benefit” favor candidates in a better clinical condition and aim to achieve better transplantation results [4]. Furthermore, not all organs and not all recipients are equal. A high-risk donor (e.g., ECD) combined with a high-risk recipient is a high-risk combination, which may qualify the transplant as futile. This has led to the avoidance of such pairings in clinical practice (risk divergence allocation policy) [5,6]. For this reason, many high-risk, waitlisted transplant candidates are penalized. To address this situation, and with the development of perfusion machines, the opposite strategy has been proposed: enable the use of marginal grafts using normothermic machine perfusion (NMP) to serve high-risk candidates (NAPLES initiative) [7].

Today, artificial intelligence (AI) is revolutionizing the field of hepatology and liver surgery. AI applications, particularly through machine learning, have now become common in the fields of diagnostic imaging and image-guided surgery [8]. Although such AI-based solutions may seem recent and novel, in 1994, Doyle et al. published the first work on artificial neural networks (ANNs) in the field of liver transplantation [9]. 

This review aims to offer a brief overview of the D–R matching crossroads. To this end, some basic AI concepts are explained, and the evolution of AI in recent years is explored to determine if this technology is a solution that seems to border on utopia.

## 2. What Is the Starting Point? The Achilles’ Heel of Traditional D–R Matching Models

**Figure 1:**
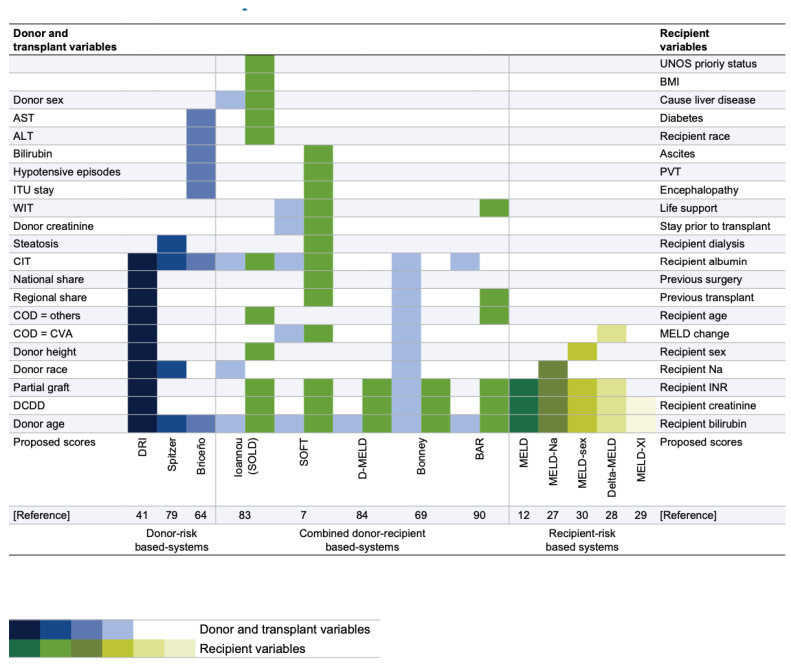
Different D–R matching systems based on donor and recipient variables. COD, cause of death; CVA, cardiovascular accident; DCDD, donation after circulatory determination of death; PVT, portal vein thrombosis. Figure obtained from Briceno J, Ciria R, de la Mata M. Donor–recipient matching: myths and realities. *J. Hepatol*. **2013**, *58* (4), 811–820. Copyright © 2022 European Association for the Study of the Liver. Published by Elsevier Ireland Ltd. All rights reserved.

The classical models (Figure 1) used to design organ allocation policies consider systems based on patient characteristics or donor risks, or a combination of donor and recipient characteristics. These D–R systems use conventional biostatistics and methodologies, such as logistic regression and other linear models [10]. Although these models have been analyzed in-depth, none offers an adequate response to D–R matching [4]. Mathematically, liver transplantation is a dichotomous problem. Different variables (donor, recipient, and logistics) are combined to obtain two possible outcomes: graft survival or graft loss at different endpoints (3 and 12 months are the most commonly used). However, no current allocation system is capable of achieving an ideal match. That is, these systems are unable to identify the candidate on the waiting list with the highest probability of death and identify, from all available grafts, the one with the highest probability of post-transplantation success for this candidate.

In allocation policies based on the sickest-first principle, the Mayo Model for End-Stage Liver Disease (MELD) score is the most commonly used score to prioritize waitlisted candidates. Over the past two decades, the MELD score has been modified several times. The current MELD score establishes a cut-off score point (<15) below which transplantation may be unsuitable for the patient and is a valid predictor of waitlist mortality. Despite its utility, however, MELD (and its modifications) shows poor predictive capacity (C-statistic of 0.55) in post-transplant survival and lacks precision in prioritizing indications other than liver dysfunction (e.g., in pediatric recipients or hepatocarcinoma) [9]. This has led to the development of special systems based on extra points [5,6,11].

Other liver scoring systems, such as the Balance of Risk (BAR) score [12] or the Survival Outcome Following Liver Transplantation (SOFT) score [13], have been validated and are being used as tools in the clinical decision-making process. The SOFT score is a reliable predictor of 3-month mortality after liver transplantation that utilizes 18 variables: 13 donor variables, 4 recipient variables, and 1 logistics variable. However, the BAR score is the best measure to predict 90-day morbidity with reasonable accuracy (area under the receiver operating characteristic [AUROC] > 0.70), as it can detect unfavorable D–R factor combinations before liver graft allocation [14]. Unfortunately, both BAR and SOFT are “all-or-nothing” scores, so they are unable to identify which of several D–R pairs will achieve the best outcome; that is, they are not “matching” systems [4].

The previous scores are all based on statistical models that include logistic regression but have important limitations [15]:

a. They assume a linear relationship between variables. Most health sciences relationships are non-linear, so this statistical methodology is not accurate.
b. The models exclude variables considered non-significant when all variables contribute to a clinical outcome to a greater or lesser degree.
c. In unbalanced problems such as liver transplantation, where deceased patients are rare, and most of them survive, logistic regression does not have an adequate predictive capacity. This is because modern biostatistics are not able to predict unbalanced phenomena, and the most common solution is to use large cohorts of patients to increase the number of infrequent events.

## 3. What Is Artificial Intelligence, and Are Machine Learning and Deep Learning the Same Concept?

AI is a branch of computational science that studies computational models capable of performing human-like activities based on two fundamental characteristics: behavior and reasoning. Its applications are diverse, including data analysis. Machine learning is defined as a branch of AI that focuses on the use of data and algorithms to mimic the way humans learn and gradually improve the algorithms’ accuracy. This learning process is understood as the ability to identify a series of complex patterns determined by a large number of variables. Therefore, the machine does not learn by itself, but the algorithm modifies itself automatically depending on the data input in its interface, thus allowing scenarios and conditions to be predicted in an automated way. For this reason, AI is being increasingly applied in the health sciences to predict clinical outcomes [16].

Machine learning can be approached in different ways, such as supervised learning (the algorithm receives already labeled data and the expected type of response), unsupervised learning (the algorithm receives an unlabeled dataset and must find its patterns), and reinforcement learning (the algorithm learns from the environment through positive or negative reinforcement). To sum up, machine learning involves the development of an algorithmic model that is then trained on data. There are many different models, such as decision tree classifiers, Bayesian networks, and neural networks (specifically convolutional neural networks). These models use a set of techniques that pursue learning through examples and are capable of recognizing complex problems and solutions, what is known as “deep learning.” Therefore, machine learning and deep learning are not on the same level, but the second is part of the first. Even so, it is possible to compare both and establish some differences.

While machine learning uses algorithms to analyze data, learn, and generate results or make decisions based on what it learns, deep learning structures the algorithms into layers of convolutional neural networks that help it learn and generate more accurate results. Data used by machine learning algorithms are structured and labeled for their predictions. This does not mean that they cannot work from unstructured data, but to do so, they need to perform some information pre-processing. Deep learning algorithms eliminate some of these pre-processing needs, as they can work with unstructured data and extract features in an automated or independent way. Finally, deep learning algorithms work in layers that reduce the margin of error. Each layer makes a judgment and combines that judgment with the result of the previous layer. The more information it receives and processes, the more accurate it becomes.

What role do deep learning algorithms play in D–R matching? As we noted in a recent publication [15], clinical decisions have both an objective and a subjective component. Scientific data, memory, and previous experiences serve as the basis for clinical reasoning, while aspects, such as intuition or emotions, form the subjective component. Therefore, clinical decisions in D–R matching have an inherent emotional bias. A single D–R matching may include around 100 parameters between the donor and recipient’s characteristics and logistical aspects. Deep-learning classifiers use multiple previous experiences based on objective data (databases) to make the best decision for which they have been trained. The subjective component of the decision is non-existent, and these classifiers are able to handle large amounts of data in a short time, which is why AI and particularly deep-learning classifiers are an interesting alternative to traditional models [17].

## 4. The Role of Deep Learning in Liver Transplantation

Deep learning provides a variety of classifiers that can be utilized in almost any field of medicine [8,16,17]. Selecting the most appropriate classifier for the problem to be studied is perhaps the greatest difficulty for those unfamiliar with how these models function. Most studies on liver transplantation have focused on the development of models to predict post-transplant graft survival. However, predicting waitlist mortality [18] or the probability of developing post-transplant acute renal failure [19] has also been the subject of study. ANNs and random forests are the most frequently used classifiers in this field, and studies aimed at improving D–R matching may use any of them.

### 4.1. Artificial Neural Networks

**Figure 2:**
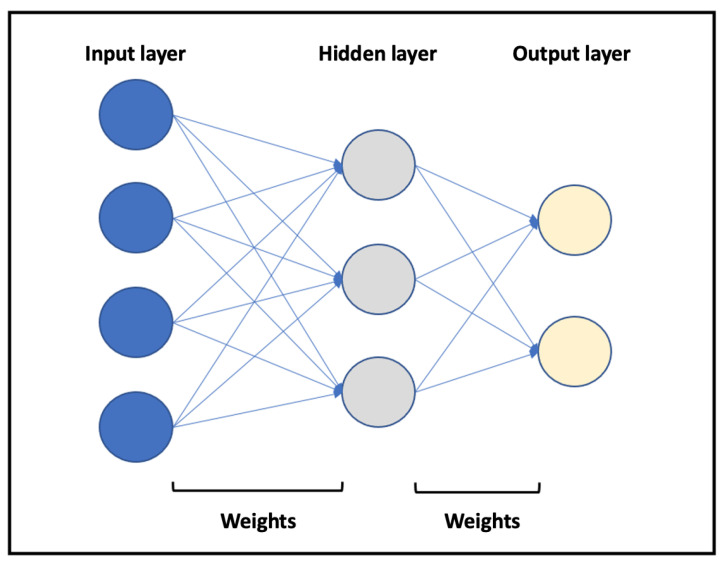
Representation of a basic neural network. Different layers are represented in blue (input layer), gray (hidden layer), and yellow (output layer). The arrows represent the relationships between neurons (weights).

The ability of ANNs to predict post-transplant outcomes based on D–R matching is promising. ANN classifiers imitate the design of human neuronal networks (Figure 2). Briefly, they consist of several groups of units (neurons) organized in different layers.

A basic neural network consists of an input layer, a hidden layer, and an output layer. The number of layers and the ANN training can vary. Both the neurons and the relationships established between them are mathematical algorithms. The relationships (weights) between the neurons in the different layers are not constant but vary as increasing data are introduced, which the model learns from. Extrapolating this to a specific clinical problem, a series of input variables is introduced into the neural network, which then processes them, according to the training received, to provide output variables of clinical interest. It is, therefore, important that the neural network is trained as robustly as possible. Briefly, the process involves splitting the dataset into two groups. The first data group is the “training set,” which includes 75% or 90% of the cases. The second group (the remaining cases) is called the “validation set” and is used to check ANN performance. Because the network is built this way, the predictive capacity (validity) of an ANN may be affected negatively by the phenomena of “overtraining” and “overfitting.”

To understand the advantages of ANNs in the field of liver transplantation, it is important to consider that the most common scenario is graft survival, while graft failure is rare, which is why it is said to be an unbalanced problem. Traditional biostatistics models are good predictors for outcomes that occur frequently; that is, they predict graft survival very well (majority class). However, they show a poor ability to predict graft failure (minority class) because it is not the usual outcome. In this regard, ANNs are able to predict both probabilities independently as they handle a large amount of data (variables). The “surviving class or majority class” prediction is based on the concept of correct classification rate (CCR, accuracy), which refers to the proportion of training patterns classified correctly by the ANN. On the other hand, the “non-surviving class or minority class” prediction capability is measured using the concept of minimum sensitivity (MS). It is necessary to understand these concepts in order to understand the results obtained by these classifiers.

**Figure 3:**
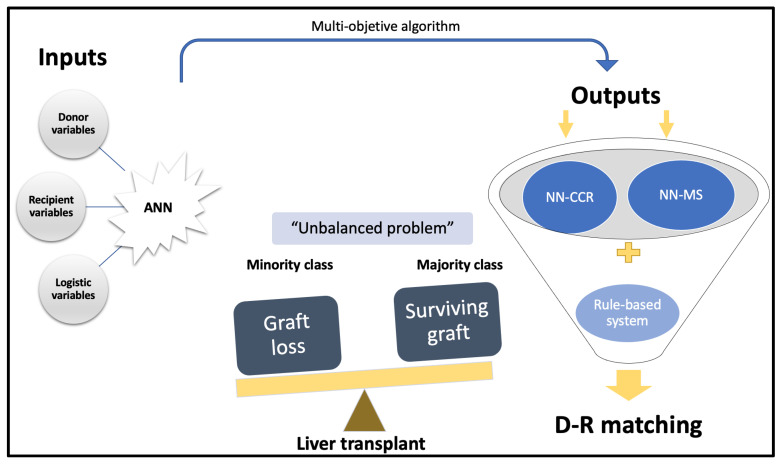
Diagram of an ANN-based on a multi-objective algorithm. Liver transplantation outcomes are shown as an unbalanced problem, and we classify them into the majority class (probability of surviving after liver transplantation, NN-CCR) and the minority class (probability of not surviving, NN-MS). By combining both probabilities (NN-CCR and NN-MS) based on input variables, we obtain a final D–R matching according to a rules-based system. ANN, artificial neural network; NN-CCR, neural network based on the correct classification rate or accuracy; NN-MS, neural network based on the minimum sensitivity.

However, since the model works with probabilities, a donor will be assigned to a recipient according to the probability of survival and graft failure without taking into account the severity of the recipient. Therefore, it is necessary to establish certain conditions for organ allocation (rules-based system). If this does not occur, the allocation would be biased, and the best candidates would receive the best grafts (i.e., those with a higher probability of success). The above concepts applied to D–R matching are schematized in Figure 3.

From a clinical point of view, Briceño et al. [20] were the first to apply a neural network combined with a system of rules to create a donor-recipient allocation model (M.A.D.R.E model). This multicenter study included a total of 1003 liver transplants performed between 2007 and 2008, using 57 variables (recipient, donor, and logistics). The probability of graft failure at 3 months was the endpoint variable. Firstly, ANN-CCR predicted a 90.79% probability of graft survival with an area under the curve (AUC) of 0.80, while ANN-MS predicted a 71.42% probability of graft loss with an AUC of 0.82. Secondly, the authors demonstrated the superiority of ANNs in donor allocation over biostatistics-based prioritization scores (MELD, D-MELD, SOFT, P-SOFT, DRI, and BAR). Finally, the allocation system used the results obtained by the constructed ANNs and successfully assigned the best candidate for a graft according to the different probabilities (CCR and MS) from among a group of patients with higher MELD, using its rules-based system.

**Figure 4:**
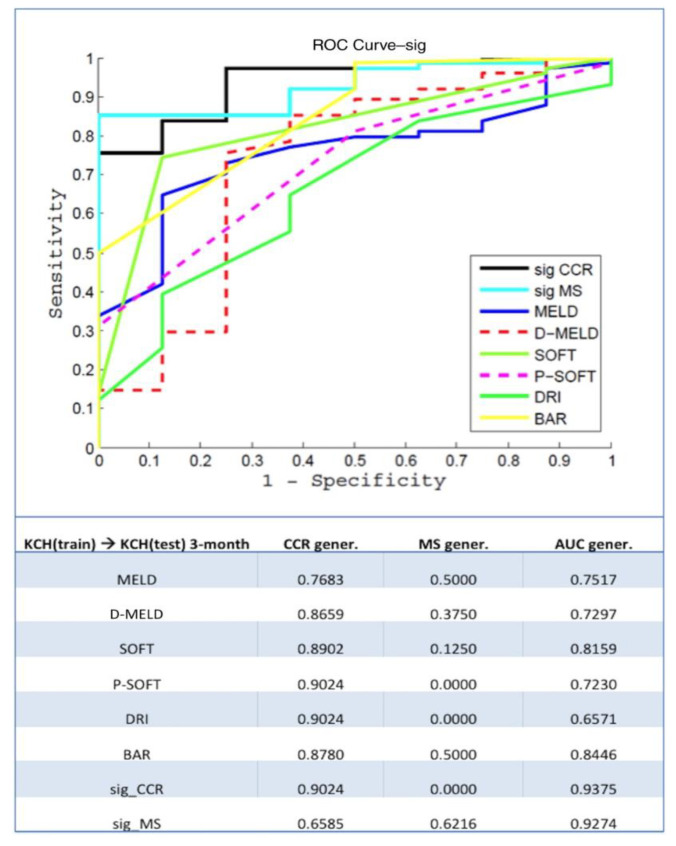
3-month graft survival model based on ANN compared to other scores using the KCH database. CCR (correct classification rate or accuracy), MS (minimum sensitivity), and AUC (area under curve) values are shown. Figure obtained from Ayllón MD et al. [21] © 2022 by the American Association for the Study of Liver Diseases.

To externally validate this methodology, a second study [21] was performed with a dataset of 858 D–R pairs from liver transplants at King’s College Hospital (KCH) in London. The authors found that the models obtained with this database achieved excellent results at 3 months [CCR-AUC 0.94; MS-AUC 0.94] and 12 months (CCR-AUC 0.78; MS-AUC 0.82). When these results were compared with other scores, such as MELD and BAR (Figure 4), a 15% difference was found in favor of the proposed model. The main reason for these findings was that a homogenous database with a low number of missing values was used. In addition to the differences in the input variables and the population, this would justify the differences between the KCH database and the Spanish model. Therefore, the authors concluded that each ANN should be used in the specific population in which it was trained.

In their most recent study, Briceño et al. [22] analyzed how ANNs work using the United Network for Organ Sharing (UNOS) dataset. This dataset comprised 39,189 liver transplants with donor, recipient, and logistics variables. Prediction of the majority class (graft survival class) and minority class (non-survival class) at different time points (3 months and 1, 2, and 5 years) were the selected endpoints. Classical statistical models (naïve Bayes or logistic regression) were compared with different machine learning models: ANN, random forest, gradient boosting, and support vector machines. For the 5-year endpoint, machine learning techniques, such as ANN (AUC = 0.599) or random forest (AUC = 0.644), were outperformed by logistic regression (AUC = 0.654). In general, the predictive capacity of the AI models (including ANNs) was very similar to that obtained by traditional models (C-statistic ≤ 0.66). The authors argued that these classifiers were trained on a database with a high percentage of missing values (only 28 variables had a percentage of less than 10%.)

#### Strengths and Weaknesses

Table 1 summarizes the main strengths and weaknesses of neural networks as a classifier. In clinical scenarios, neural networks are very useful for finding patterns that are far too complex or numerous since they can generate near-perfect predictions using the data on which they are fit [23]. In addition, data processing is performed quickly—an essential aspect of graft allocation. However, ANNs are inherently opaque and lack interpretability because the set of weights or algorithms in hidden layers is unknown. This is called the “black-box” issue, which has made many clinicians skeptical of their use because it is necessary to know all the details of the process [8].

The predictability of an AI model depends on the robustness of the database. Consequently, ANNs for D–R matching can only be applied in very homogeneous databases that follow similar rules for the prioritization and inclusion of candidates. This is why ANNs may work very well in local and regional liver transplantation programs but cannot be extrapolated to other centers, thus requiring regional-specific ANN models.

Although the results of predictive models are good, most databases are small, have a high number of missing values, or can only be applied to the population where the ANN has been trained. In addition, neural networks depend on a system of rules to properly perform donor–recipient matching but are based on the sickest-first principle. Therefore, nowadays, ANNs can only assist, but not carry out, the matching decision in all aspects of organ transplantation [24,25].

### 4.2. Random Forests

Random forests are deep-learning classifiers based on decision trees. It is an ensemble-type methodology (i.e., a model of models), and it is necessary to determine the number of models that will form the final model and verify that these models are not correlated because their results will not be very adequate. Aside from these two drawbacks, the methodology is superior to the decision trees from which it comes. For each of the possible outputs, a different decision tree is built. The database is “split” by the researcher into different nodes. The database requires a previous treatment filter to avoid over-training and overfitting the data.

Lau et al. [26] examined how models based on random forests could predict post-transplant graft failure compared with other scores. The dataset included 180 liver-transplanted patients (173 donor and 103 recipient variables). Random forests and ANNs were compared with the Donor Risk Index (DRI), MELD, and SOFT. The random forest demonstrated its superiority with an AUC of 0.787 compared to ANN (AUC = 0.734) and DRI (AUC = 0.595). In addition, they obtained a simplified model of 15 variables that achieved an AUC of 0.715. The percentage of missing values in these 15 top variables ranged from 0% to 72.22%, with 5 variables with missing values >10%, thus demonstrating the ability of random forest models to work with a high percentage of incomplete data.

#### Strengths and Weaknesses

The most important advantages of random forest classifiers are that (a) it is not necessary to normalize the variables, unlike neural network models, which require normalization of the independent variables of the model; (b) unlike ANNs, they perform very well with a small database and a high percentage of missing values [27]; and (c) they have excellent predictive power, thus providing a very nice and sophisticated output with variable importance.

Unfortunately, these models are not useful with larger datasets since the number of decision trees they generate can be unmanageable. When looking for the minority endpoint in an unbalanced problem (i.e., graft failure in liver transplantation), a larger database is required to have more casuistry. Thus, the model accuracy may be affected. Moreover, they have a high risk of “over-fitting,” that is, their effectiveness on the training dataset is sometimes much higher than that obtained on the validation and/or generalization dataset.

### 4.3. Study Limitations

The implementation of AI in the field of liver diseases has grown exponentially. However, the number of clinical (not methodological) papers addressing D–R matching is small. Most of the papers mentioned are observational studies (data retrieved from databases). To truly test the predictive power of artificial intelligence, large prospective cohort studies with external validation are needed. To date, only one author has validated this methodology externally [21]. However, external validation may be questionable since classifiers based on deep learning perform better in populations where they are trained. Thus, the most realistic and suitable option would be to use region-specific models [15].

Given that the studies differ significantly in terms of size, design, prediction models, and dataset quality, it is difficult to determine which classifier is preferred for each scenario. Finally, a time-to-event (i.e., graft failure) approach could be interesting as this has not been done to date. This approach could be useful as a quantitative measure of survival gained/lost when accepting/rejecting a specific organ, which would aid clinicians when making decisions about grafts [28].

From an ethical point of view, there are three barriers to overcome. The first is the “black box issue,” which may cause mistrust among clinicians because they do not know the weight of the variables in the models. The second is data privacy and cyber security. The last barrier is finding an adequate answer to the following question: Who is responsible if the model fails?

## 5. Conclusions: What Is on the Horizon?

AI has contributed to the field of liver transplantation through different classifiers, such as ANNs or random forests [29]. On the one hand, machine learning classifiers operate impartially as they are not affected by subjective factors. On the other, they can handle a multitude of variables of clinical interest in a quick and easy way (faster than humans) to identify the best outcome. These are the main reasons that make the use of AI so attractive from a clinical point of view. Achieving better outcomes in liver transplantation implies a lower economic investment compared with dysfunctional grafts. Additionally, better D–R matching would lead to better post-transplant outcomes that would be more cost-effective in the long term than dysfunctional grafts. Methodologies such as AI that aim to improve D–R matching in these terms would also reduce procedure costs, thus obtaining individual and social benefits [30].

Deep learning is the branch of artificial intelligence that appears to be undergoing the greatest development in this field. Nitski et al. [31] assessed the ability of deep-learning algorithms to predict post-transplant complications that result in patient death. The AUCs for prediction of death by graft failure within one year achieved by ANN models (with a low missing values dataset) ranged from 0.847 to 0.871, values that were replicated in the testing group. Other examples include the application of deep learning to assess CT volumetry in living donors [32], the prediction of hepatocellular carcinoma recurrence after liver resection [33], or the identification of hepatic steatosis in living donors [34].

However, while the scientific production related to AI is more abundant in other areas of liver disease [8], D–R matching remains controversial. The works published to date show interesting results but have been unable to achieve clinical applicability. In the opinion of the authors, there are three key points to implement these models in our clinical decisions: (a) overcome our skeptical mentality and ethical barriers as clinicians; (b) collect data without missing values to build large and robust datasets since robust datasets lead to accurate models and confidence models; (c) do not consider AI-based tools as “self-driving cars,” but as tools to support decisions and complement current systems.

## Figures and Tables

**Table 1:** Main strengths and weaknesses of neural networks as a classification method based on artificial intelligence.

Artificial Neural Networks. Strengths and Weaknesses
*Strengths* Ability to work with incomplete knowledge. After ANN training, the data may produce output even with incomplete information. Fault tolerance. The corruption of one or more neurons of the ANN does not prevent it from generating output, even if it reduces the training capacity of the network. A distributed memory. For an ANN to be able to learn, it is necessary to determine the examples of a database and teach the network according to the desired output by showing it these examples. Ability to make machine learning. With neural networks, classification, clustering, and regression models associated with decision support systems are built. Parallel processing capability. Artificial neural networks have numerical strength that can perform more than one job at the same time. They are applied in many branches of knowledge: medicine, engineering, economics, agriculture, energy, climate change, ecology, etc.
*Weaknesses* Hardware dependence. ANNs require processors with parallel processing power in accordance with their structure. Therefore, current convolutional neural networks require GPU graphics cards. Unexplained behavior of the network. The models obtained with this methodology lack the ability to interpret the results obtained. It is one of the main disadvantages of this type of methodology. Determination of proper network structure. There is no specific rule for determining the structure of ANNs. Thus, if an adequate architecture is not designed, the results can be approximate. Difficulty of showing the problem to the network. ANNs work with numerical information, so other types of information must be transformed into numerical information.

## Data Availability

Not applicable.
